# Construction-induced soil compaction in agri-photovoltaic systems: evidence from an Arenosol

**DOI:** 10.1038/s41598-026-65268-z

**Published:** 2026-08-16

**Authors:** Kathrin Grahmann, Tatiana Reis dos Santos Bastos, Marco Donat, Lina Rohlmann, Moritz Reckling

**Affiliations:** 1 Resource-Efficient Cropping Systems, Research Area Land Use and Governance, Leibniz-Centre for Agricultural Landscape Research (ZALF), Eberswalder Str. 84, 15374 Müncheberg, Germany; 2 Departamento de Engenharia Agrícola e Solos, Universidade Estadual do Sudoeste da Bahia – UESB, Vitória da Conquista, Vitória da Conquista, BA Brazil; 3 Department of Landscape, Society, and Economy, Eberswalde University for Sustainable Development (HNEE), Schicklerstraße 5, 16225 Eberswalde, Germany; 4 Provisioning of Ecosystem Services in Agricultural Systems, Land Use and Governance, Leibniz-Centre for Agricultural Landscape Research (ZALF), Eberswalder Str. 84, 15374 Müncheberg, Germany; 5 Institute of Earth System Sciences, Section Soil Science, Leibniz University Hannover, 30419 Hannover, Germany; 6 Department of Crop Production Ecology, Swedish University of Agricultural Sciences (SLU), Uppsala, 75007 Sweden

**Keywords:** Bulk density, Penetration resistance, Sandy soil, Spatial diversification, Solar energy, Soil moisture, Engineering, Environmental sciences

## Abstract

Agri-photovoltaic (Agri-PV) systems, which combine crop production with solar energy generation, are rapidly expanding across Europe, yet their implications for soil quality remain poorly understood. This study assessed soil compaction following the installation of an Agri-PV system in autumn 2024 on an Arenosol in Brandenburg, Germany. Bulk density (BD) and penetration resistance (PR) were measured across multiple depths after construction finalization. Both parameters were compared to a nearby reference and established agronomic thresholds. Subsoil BD in construction zones (1.67–1.69 g cm⁻³) exceeded root growth thresholds, while PR at mid-depth reached 3.7–4.1 MPa, surpassing the limit for root elongation. Our results align with literature indicating that Arenosols, due to their weak structure and lack of shrink-swell capacity, are highly vulnerable to persistent compaction. The observed compaction poses risks to rooting depth, water infiltration, and crop yield, underscoring the need for targeted mitigation measures. We suggest that pedological construction supervision should become standard practice for Agri-PV projects, ensuring soil protection measures are integrated into planning, construction, and restoration. More flexible policy frameworks and monitoring of soil recovery post-installation are needed for aligning renewable energy expansion with sustainable crop production.

## Introduction

Agri-photovoltaic (Agri-PV) systems, which combine agricultural production with solar energy production, are rapidly expanding across Europe as part of renewable energy strategies, making them an essential component for climate change mitigation^1,2^. In Germany, expansion targets increasingly focus on co-utilized areas, including Agri-PV systems, parking lots and floating systems to reduce land-use competition^3^. Agri-PV systems align with sustainable agricultural transformation goals, as they offer potential synergies between food production, renewable energy generation, and biodiversity conservation^4^ while enhancing societal acceptance of renewable energy. The establishment of integrated production and distribution systems within Agri-PV is expected to unlock systemic innovations, moving these systems from niche to mainstream applications^5^.

Agri-PV systems alter microclimatic production conditions such as radiation, temperature, and soil moisture, which can affect crop yields depending on site conditions and crop type^6–10^. These subsequent effects during system operation must be clearly distinguished from soil disturbance occurring prior during the construction phase. While most research has focused on agronomic effects of running Agri-PV systems, soil disturbance during construction and its long-lasting impacts on soil physical functioning remain largely unquantified. A significant trade-off, particularly regarding soil health (here understood as soil physical functioning^11^) during and after installation has been reported already for common solar panel power plants installed over soils that are left fallow^12^. The Agri-PV construction process involves the use of heavy machinery for material transport and structural installation, which may contribute to soil compaction. Soil degradation due to soil compaction is a continuously growing threat by heavy agricultural machinery used for all sorts of field interventions and crop production steps^13^^,14^. Compaction reduces soil porosity and hydraulic conductivity, restricting root growth and water infiltration^15,16^. These soil processes are particularly critical in agricultural systems, where healthy, non-degraded soils contribute to both crop productivity and ecosystem functioning, such as carbon storage and water cycle regulation^17,18^.

The degree of soil compaction caused by farming as well as construction machinery is strongly controlled by soil type, moisture content, and traffic frequency. Coarse-textured soils like Arenosols with low organic matter and buffering capacity exhibit lower structural stability and limited water retention due to their larger particles and pore spaces^19^. This makes them less reactive to compaction compared to finer-textured silty or clayey soils like Luvisols, which are more vulnerable when moist^20^. However, once compacted, sandy soils have limited ability for spontaneous recovery and require alleviation and amelioration measures^21^. These contrasting responses highlight the importance of pedological context when assessing construction-related soil impacts in Agri-PV systems. Key indicators of soil compaction include bulk density and porosity, which are strongly related to soil structure and soil moisture in soils with shrink-swell properties^22,23^. The upper soil layers are mostly affected by compaction, which directly inhibits root growth during early crop establishment^13,24^. Another relevant compaction parameter is soil penetration resistance, which informs about the vertical distribution patterns of compacted soil layers and depends on bulk density, soil texture and soil moisture ^25,26^. Despite the rapid expansion of Agri-PV across Europe, empirical studies quantifying soil compaction caused by installation activities remain extremely limited.

Efforts to mitigate construction-induced compaction in Agri-PV systems emphasize adopting best practices such as using lighter machinery and/or mobile construction roads, avoiding high-traffic zones during installation, and scheduling the construction during periods of low soil moisture and thus precipitation^27,28^. However, economic constraints, logistical challenges, and policy-driven timelines often limit the consistent implementation of these measures. To date, experimental studies have primarily reported post-installation soil effects in common PV facilities, including spatial heterogeneity in soil moisture and unsaturated hydraulic conductivity due to the photovoltaic array^29^, as well as reduced mean weight diameter of soil aggregates and porosity following solar park construction^30^.

Recent reviews highlight the limited understanding of soil physical responses and pedogenic processes under Agri-PV systems, particularly regarding construction-induced impacts and their spatial heterogeneities to detect main drivers for future crop development and yield performance^31–33^. This study addresses the knowledge gap on construction-induced soil compaction in Agri-PV systems. We hypothesize that heavy machinery traffic during installation leads to spatially heterogeneous but persistent soil compaction in an Arenosol exceeding critical thresholds for root growth. For this, we aimed to quantify bulk density and penetration resistance across depths and locations within an Agri-PV system and relate these patterns to potential implications for soil functionality and crop production. This case study provides transferable insights into soil risks associated with Agri-PV expansion under comparable pedological and climatic conditions.

## Results

### Soil properties

The soil analysis showed considerable variation in both physical and chemical properties across the experimental site (Table 1). Higher carbonate (TIC) concentrations were observed in the second and fifth depth increments, contributing to total carbon content in the Agri-PV site. Organic carbon levels above 1% were found only in one sample (D2, 12–17 cm depth) in transect 2, suggesting past accumulation that may have been redistributed to deeper layers, possibly due to plowing during site preparation. The control reference site also contained one sampling point in the topsoil (D1) of transect 4 with 1.38% TOC which aligns with its long fallow history and organic matter accumulation. Soil texture varied but was predominantly sandy, with occasional clay layers at two points of transect 3 in 20 to 50 cm depth containing up to 15–17% clay, and a pronounced silty layer in 20–25 cm at one of the control sites in transect 2 (Table 1). Higher soil pH above 7 was found in samples containing carbonates, particularly in deeper layers, while topsoil samples generally resulted in lower pH levels.

**Table 1:** Physical and chemical soil properties at the experimental site (Ct: total carbon in %, Nt: total nitrogen in %, TIC: total inorganic carbon in %, TOC: total organic carbon in %, TC: total carbon as sum of TIC & TOC in %, pH: Soil pH with KCl extraction, Clay, silt and sand content in %, KA5: Bodenkundliche Kartieranleitung (Ad-Hoc-AG Boden^34^; German soil textural classes: Su2 = slightly silty sand, Su3 = medium silty sand, Su4 = strongly silty sand, Sl2 = slightly loamy sand, Sl4 = strongly loamy sand), θ₃₃ (FC, %): estimated field capacity* at pF ≥ 1,8; % SM_grav_: gravimetric soil moisture content at bulk density sampling in %; BD_corr_: stone-corrected bulk density in g cm^-3^; 2D: 2D tracker system, Con: control reference site) across four selected transects (T1-T4) and five depths (4–9 cm (D1), 12–17 cm (D2), 20–25 cm (D3), 45–50 cm (D4) and 70–75 cm (D5). Figure 8D depicts the random distribution of sampling points for chemical analysis. na: not available due to low amount of soil sample mass, *high stone content.

System	Transect	Depth	% Ct	% Nt	% TIC	% TOC	% TC	pH (KCl)	Clay	Silt	Sand	KA5	θ₃₃ (FC, %)	% SM_grav_	BD_corr_
2D	T1	D1	0.95	0.076	0.009	0.94	0.95	5.6	3	22	75	Su2	24.7	17.9	1.67
	T2	D1	0.60	0.052	0.0055	0.59	0.59	5.0	3	22	75	Su2	22.9	12.7	1.76
	T3	D1	0.92	0.082	0.0107	0.91	0.92	6.5	6	21	72	Sl2	27.4	12.2	1.91
	T2	D2	1.74	0.137	0.0168	1.70	1.71	6.3	3	21	76	Su2	23.6	14.8	1.40
	T3	D2	0.77	0.050	*0.3065*	0.46	0.77	*7.7*	na	na	na	na	na	11.1	1.82
	T3	D3	0.26	0.032	0.0	0.22	0.22	5.7	17	21	62	Sl4	35.6	13.0	1.91
	T1	D4	0.18	0.016	0.0004	0.16	0.16	5.8	3	22	75	Su2	23.1	16.2	1.63
	T3	D4	0.22	0.026	0.0	0.19	0.19	5.7	15	23	63	Sl4	34.2	12.7	1.86
	T1	D5	0.14	0.013	0.0	0.11	0.11	5.4	1	18	81	Su2	21.4	na	0.86*
	T4	D5	0.14	0.016	0.0	0.11	0.11	5.3	6	34	60	Su3	21.0	13.9	1.94
	T4	D5	1.20	0.010	*1.0548*	0.08	1.14	*7.9*	6	29	66	Su3	20.6	10.8	1.99
CON	T4	D1	1.47	0.129	0.0216	1.38	1.40	6.6	3	25	72	Su2	25.7	20.9	1.18
	T4	D2	1.05	0.091	0.0127	1.00	1.01	5.5	4	25	72	Su2-Su3	24.8	15.5	1.21
	T2	D3	0.51	0.045	0.0046	0.49	0.50	5.6	7	48	45	Su4	31.8	8.9	1.44
	T1	D4	0.16	0.010	0.0	0.11	0.11	7.3	2	20	78	Su2	22.2	21.1	1.08

### Soil moisture

**Fig. 1:**
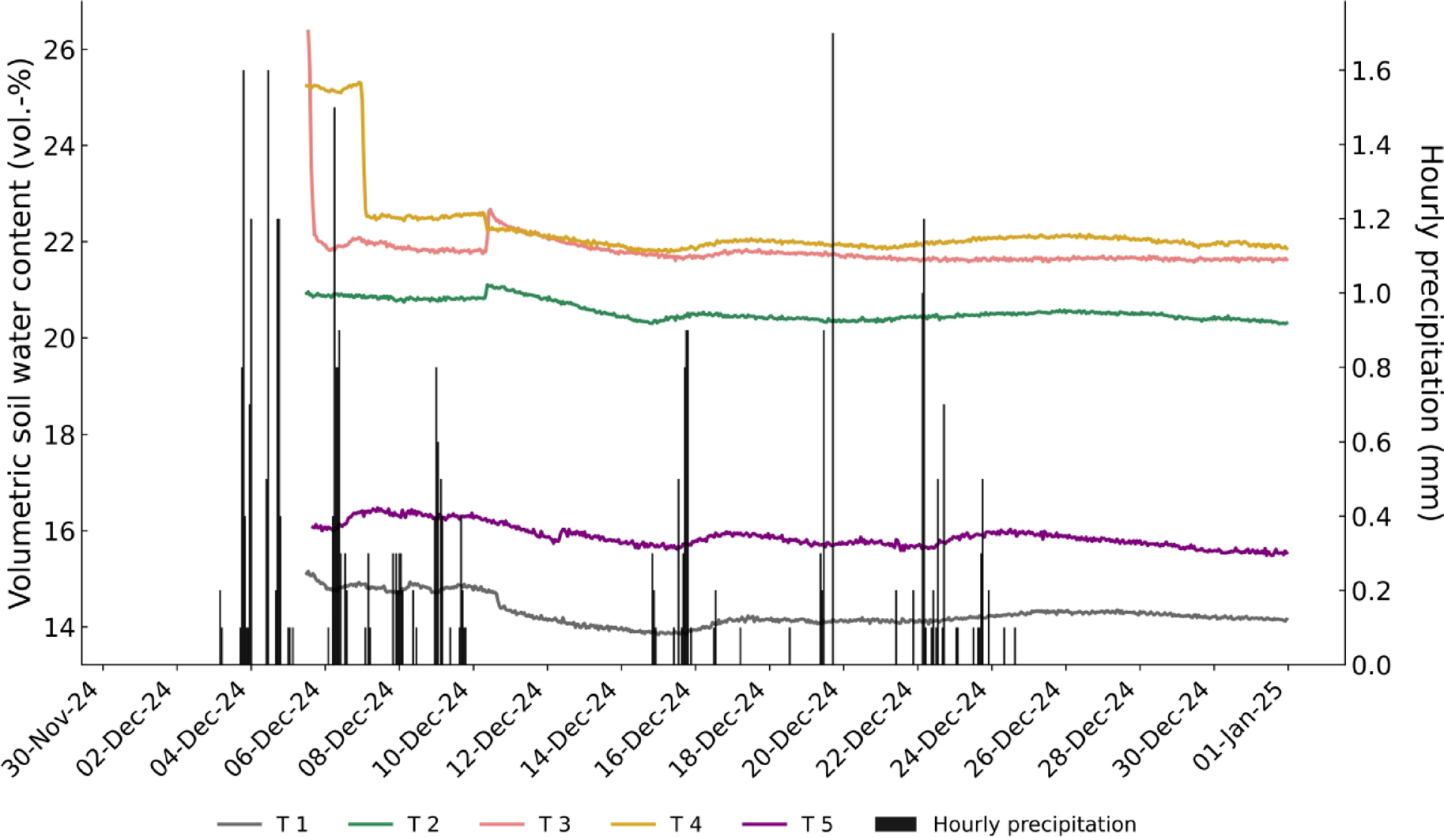
Hourly volumetric soil water content (vol.-%) averaged for three centric sensors per transect (T1 to T5, compare location in Fig. 8D) at a depth of 45 cm and hourly accumulated precipitation in mm in December 2024.

Soil moisture was spatially heterogeneous across the transects (Fig. 1). Volumetric soil moisture in T2, T3 and T4 consistently exceeded 20%, whereas moisture in T1 and T5 remained below 17%. Volumetric soil moisture in T3 and T4 resulted in a sharp drop shortly after sensor installation on 5th of December. Soil moisture levels remained relatively stable from mid-December onwards, with only gradual declines likely reflecting drainage under low temperatures and absence of vegetation. However, pronounced moisture differences between transects may also have contributed to penetration resistance pattern measured on 17th of December with T1 having only around 14%, followed by T5 with 16%, T2 with 20.5% of soil moisture and T3 and T4 having 22% each (Fig. 1). Overall, the recorded values showed generally moist towards saturated soil conditions in 45 cm depth after the construction phase ended. Gravimetric soil moisture obtained during bulk density sampling on 25th November showed lower, but less variable moisture levels across depths, with values at 45–50 cm ranging between 11% in T3 and 15% in T2. More variable gravimetric soil moisture between 10% (T5) and 23% (T1) was measured in 75 cm depth, stating generally moist, but not fully saturated subsoil conditions (data not shown). Combined with precipitation totals of 52.6 mm in November and 40.6 mm in December and low average temperatures (< 5 °C), the measurements indicate generally moist subsoil conditions during the sampling period, but mostly below estimated field capacity (between 20 and 25 vol.-% for the slightly silty sand soil type, Table 1).

The observed variability in soil texture, nutrient concentration and soil moisture content indicates a heterogeneous site, which may contribute to spatial variability in bulk density and penetration resistance alongside construction-related effects.

### Bulk density

**Fig. 2:**
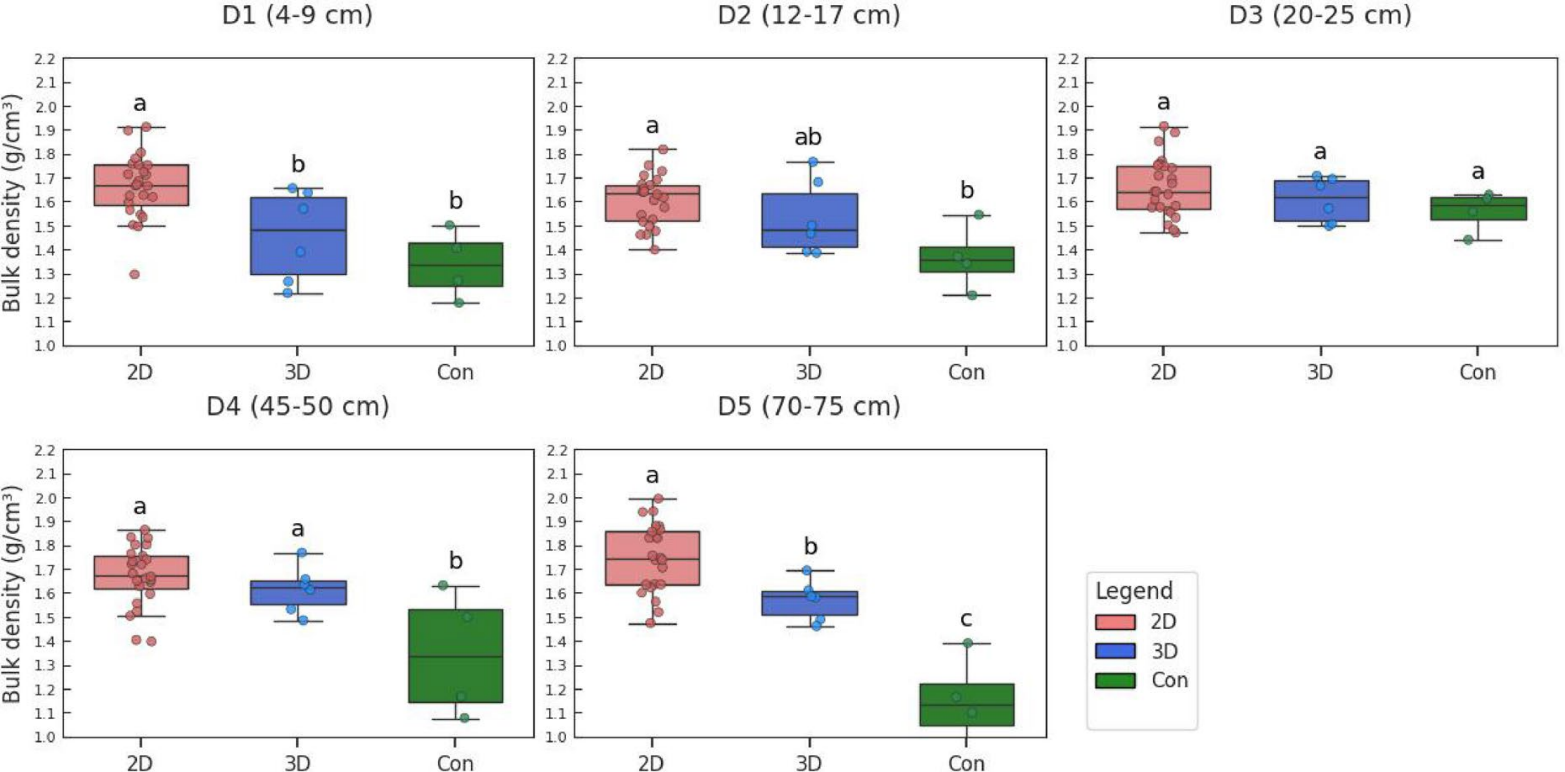
Bulk density (in g cm^−^³) at five depth intervals (D1: 4–9 cm, D2: 12–17 cm, D3: 20–25 cm, D4: 45–50 cm, D5: 70–75 cm) averaged for each agri-photovoltaics system (2D, 3D) and the control site (Con). Different letters indicate significant differences between systems (2D (*n* = 24), 3D (*n* = 6), Con (*n* = 4)) by Tukey test (*p* < 0.05) per depth layer. Note: agricultural (Ag) and pole (P) locations have been averaged in both Agri-photovoltaics systems.

Bulk density was significantly higher in 2D and 3D Agri-PV systems compared to the control site (Con) in four out of five depth intervals, with no significant differences observed between systems in the 20–25 cm depth interval (D3) (Fig. 2). In the subsoil, the control site exhibited significantly lower bulk density values, averaging 1.34 to 1.14 g cm^−^³ in D4 and D5, respectively, while bulk density under both Agri-PV systems remained consistently high, averaging 1.67 to 1.69 g cm^−^³ at 70–75 cm depth. Individual sampling points reached harmful levels of 1.86 to 1.99 g cm^−^³ in the Agri-PV subsoil.

**Fig. 3:**
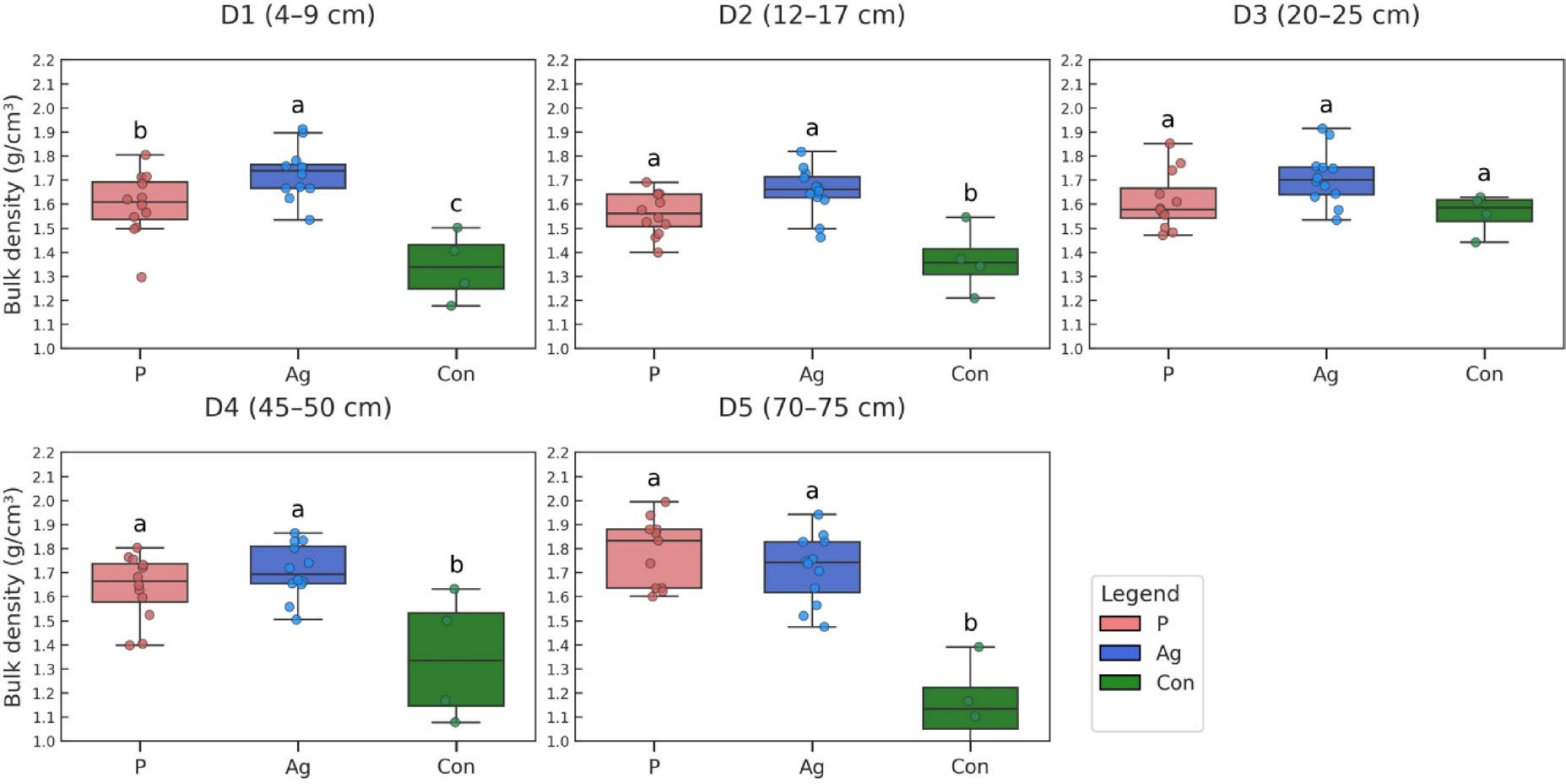
Bulk density (in g cm^−^³) at five depth intervals (D1: 4–9 cm, D2: 12–17 cm, D3: 20–25 cm, D4: 45–50 cm, D5: 70–75 cm) averaged over four transects in pole (P), agricultural (Ag) and control (Con) locations in the 2D system. Different letters indicate significant differences between locations (P (*n* = 12), Ag (*n* = 12), Con (*n* = 4)) by Tukey test (*p* < 0.05) per depth layer. Note: data from the single 3D transect has been excluded.

Bulk density was significantly higher at both the pole (P) and agricultural (Ag) locations compared to the control site, with most pronounced differences observed in the subsoil layers (D4 and D5), but not in the intermediate layer (D3) (Fig. 3). No statistically significant differences were detected between P and Ag zones except in the topsoil, indicating a similar level of compaction below 9 cm soil depth across the entire 2D Agri-PV system. Values smaller than 1.4 g cm^−^³ indicate no subsoil compaction and were almost exclusively found at the control site.

Across all five transects in 2D and 3D systems (excluding control site samples), bulk density values ranged from 1.30 to 1.99 g cm^−^³ across all depths, indicating a broad soil density spectrum within the Agri-PV site. Median values exceeded 1.6 g cm^−^³ in most transects (data not shown), particularly in deeper layers, suggesting a moderate to high level of subsoil compaction. Depth alone did not explain the observed variability in bulk density; however, variability was highest in D3 and D4 and less pronounced in D5.

### Penetration resistance

**Fig. 4:**
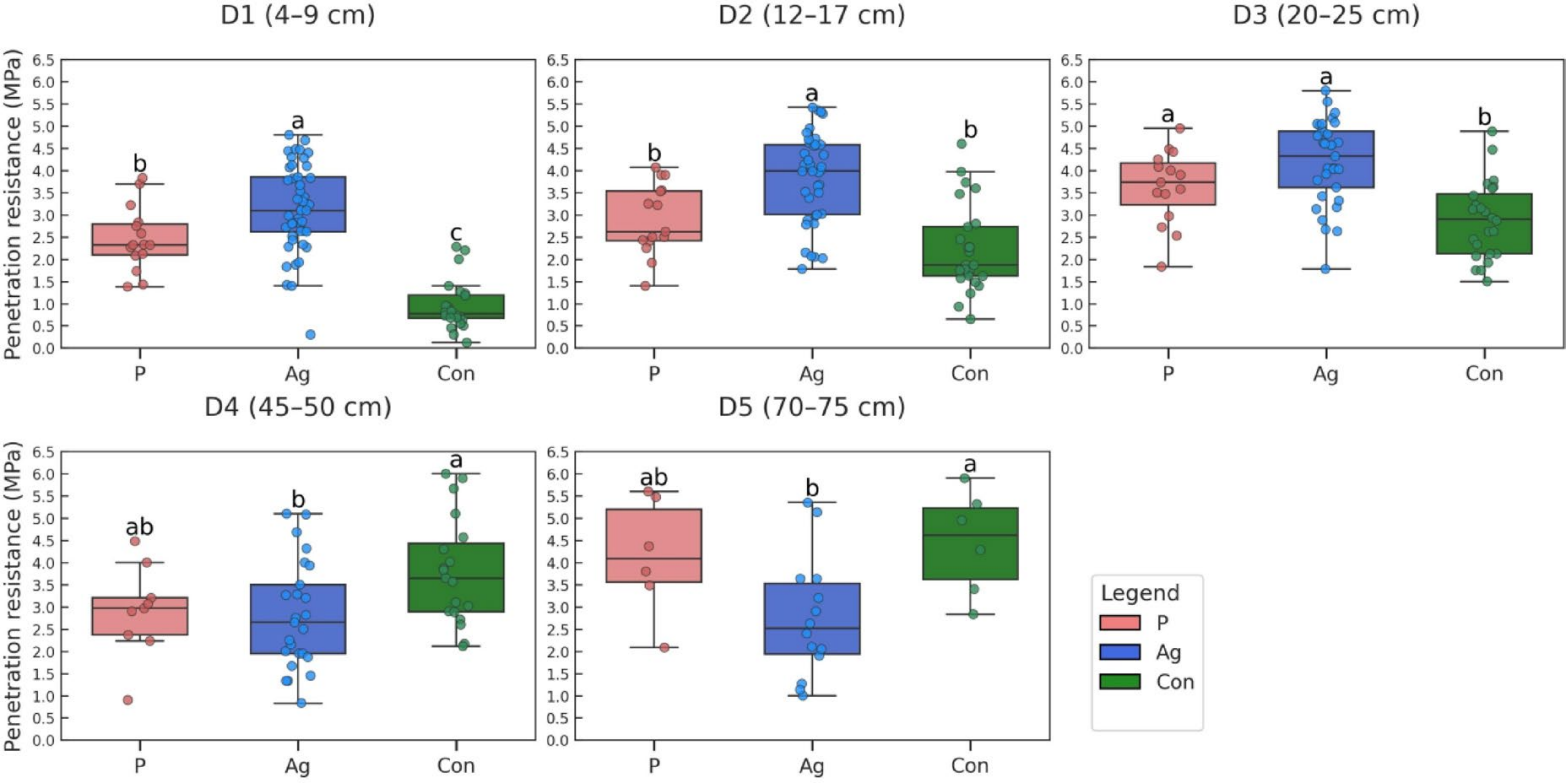
Soil penetration resistance (in MPa) at five depth intervals (D1: 4–9 cm, D2: 12–17 cm, D3: 20–25 cm, D4: 45–50 cm, D5: 70–75 cm) averaged over five transects in pole (P), agricultural (Ag) and control (Con) locations within the 2D system. Different letters indicate significant differences between locations (P (*n* = 15), Ag (*n* = 45), Con (*n* = 25) in D1) by Tukey test (*p* < 0.05) per depth layer. Note: data from the 3D transects are not included.

The highest average penetration resistance was recorded in the 20–25 cm depth interval (D3), reaching 4.1 MPa at agricultural (Ag) locations and 3.7 MPa near the pole (P) positions (Fig. 4). In contrast, the control site exhibited its highest average resistance of 4.7 MPa in the deepest layer (D5, 70–75 cm). However, it is important to note that the number of valid observations declined with increasing soil depth. This was primarily due to technical limitations of the penetrologger: in highly compacted subsoil layers, the device was either unable to fully penetrate the soil or returned negative, erroneous values due to excessive resistance. As a result, sampling points with extreme compaction, where penetration resistance likely exceeded critical thresholds, were systematically excluded from the dataset. This limitation suggests that actual compaction levels, particularly in the deeper layers under Agri-PV, may be underestimated in the present study. Thus, bulk density measurements in the subsoil provide complementary evidence of compaction, particularly in those layers where penetration resistance measurements were limited.

Penetration resistance was elevated in the Agri-PV locations, particularly in the topsoil (D1) and mid-depth (D3) layer, while differences were less pronounced in D2. Higher penetration resistance was not confined to structural site elements (e.g., poles) but extended into the agricultural production area, likely as a result of frequent construction traffic between the tracking rows during the installation of the panels. In the subsoil layers (D4 and D5, 45–75 cm), no significant differences were observed between locations. However, median values in D4 and D5 were highest at the control site (Fig. 4), possibly due to pre-existing site conditions but also caused by exclusion of high (negative) resistance data from Ag and P locations. Among all locations, the agricultural area showed the greatest variability in penetration resistance, as indicated by wide ranges between minimum and maximum values. This suggests spatially heterogeneous compaction, potentially caused by uneven machinery movement or localized differences in soil texture and moisture conditions during the construction phase.

**Fig. 5:**
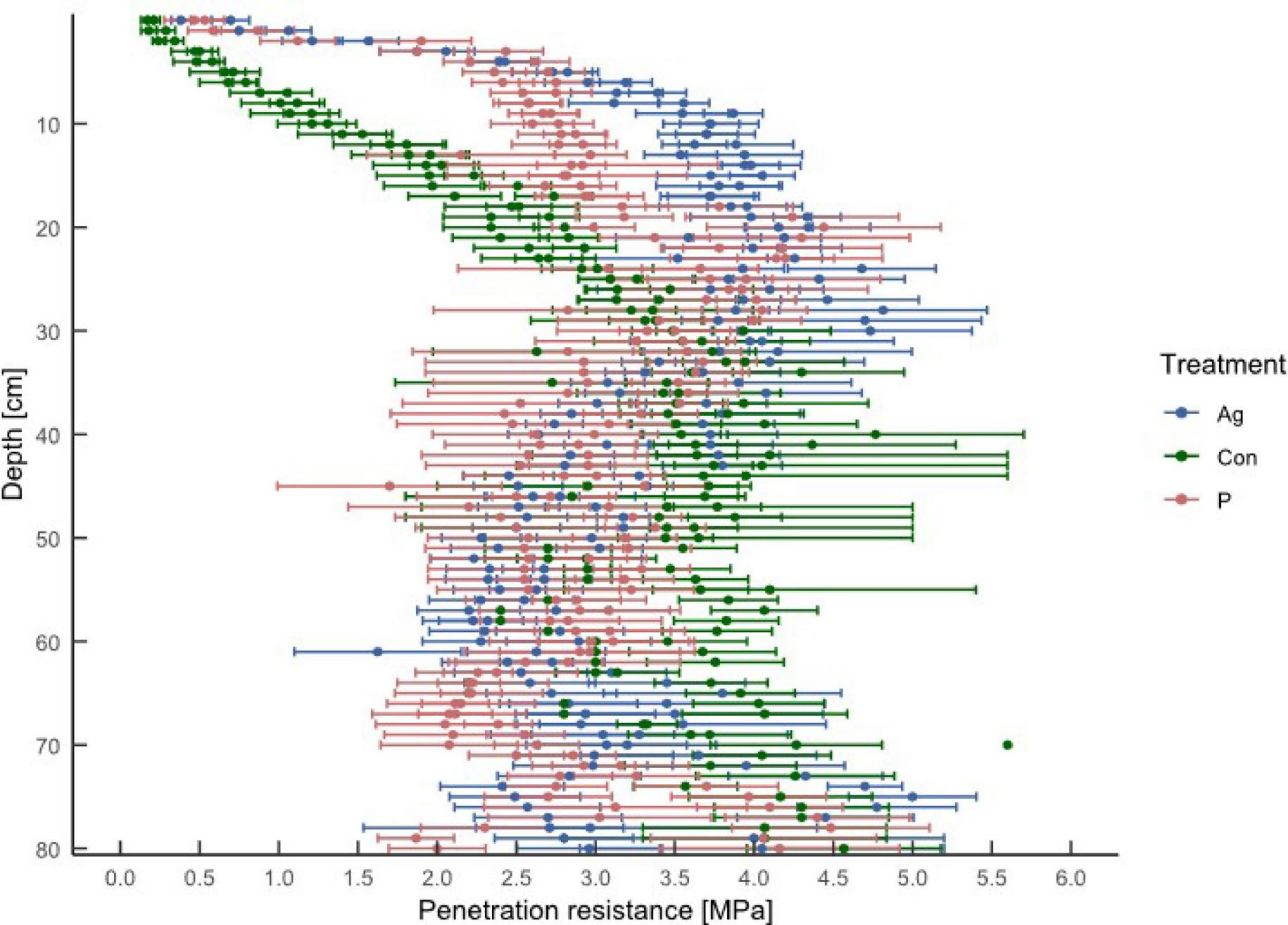
Depth profile of soil penetration resistance (in MPa) measured in 1 cm increments down to 80 cm depth. Data represent averaged values of seven transects across both Agri-PV systems (2D and 3D) at pole (P), agricultural (Ag) and control (Con) locations. Dots and horizonal lines indicate the mean ± standard error SE.

The centimeter-wise soil profile revealed substantially lower penetration resistance at the control sites (Con) up to a depth of 30 cm, followed by a pronounced increase in resistance with depth (Fig. 5). In contrast, the agricultural sampling locations exhibited the highest penetration resistance values within the upper 30 cm, suggesting surface and near-surface compaction likely caused by machinery traffic during Agri-PV construction. At greater depths, the control site showed the highest penetration resistance, pointing to pre-existing subsoil compaction unrelated to the Agri-PV installation. Spatial variability between transects (SE in Fig. 5) was low in the top 30 cm but increased notably with depth. Again, the number of valid observations decreased in deeper layers due to erroneous readings from the penetrologger, which failed to penetrate highly compacted soil or exceeded its operational limits.

**Fig. 6:**
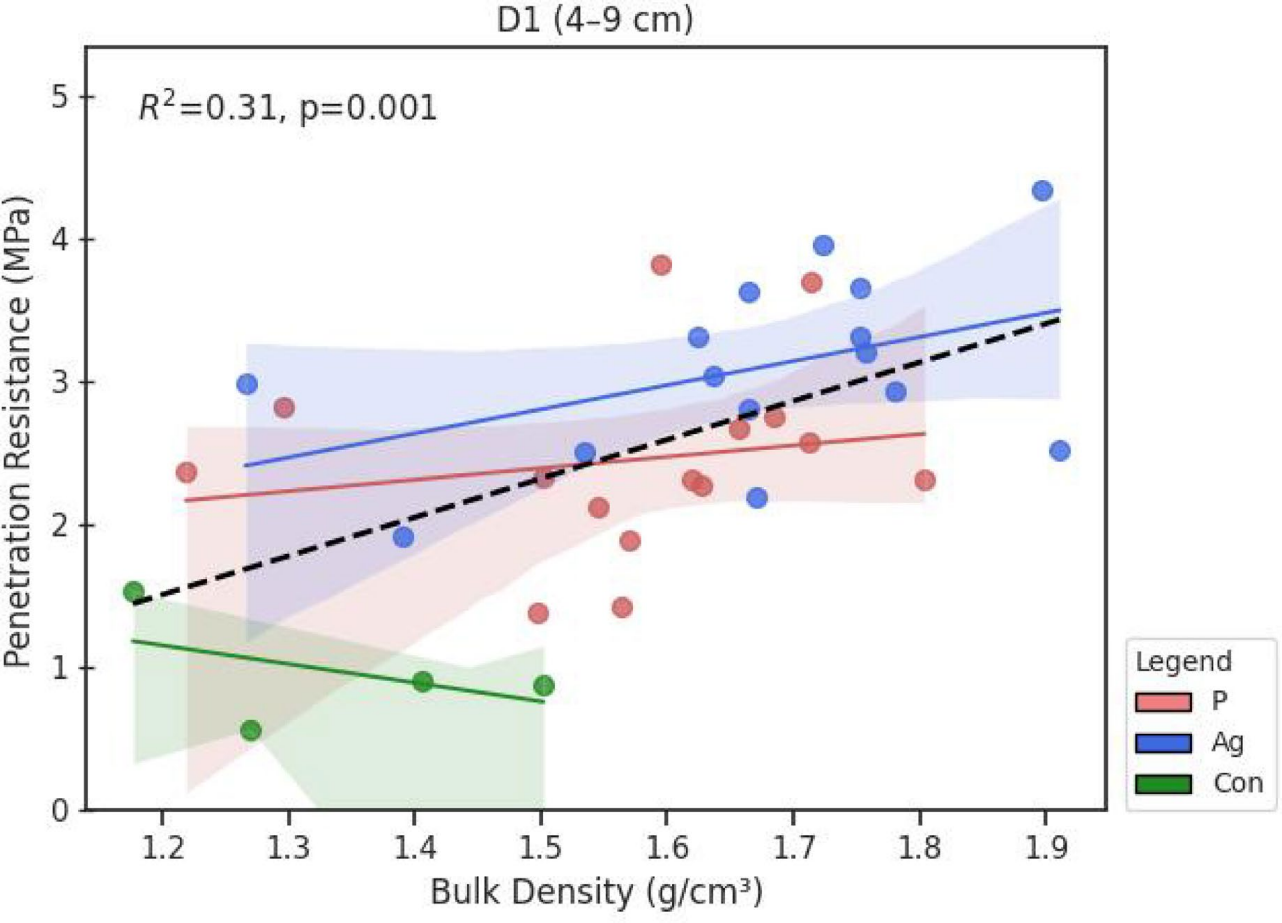
Regression analysis between data pairs of penetration resistance and bulk density obtained from five transects, three different locations and both Agri-PV systems, considering depth D1 from 4–9 cm, where a significant, but weak relationship was observed.

A slight positive significant relationship between bulk density and penetration resistance was only observed in the first depth interval (D1) (Fig. 6). However, due to the limited number of data pairs available for the control sites (maximum of four), no robust individual correlation analysis could be performed for this location.

## Discussion

### Bulk density exceeds established compaction thresholds

Bulk density (BD) values obtained at the Agri-PV site clearly exceeded those of the control site, representing pre-construction conditions. BD values of the control averaged 1.34–1.56 g cm^−^³ in the first three layers, whereas values in the Agri-PV systems ranged from 1.62 to 1.65 g cm^−^³. This is comparable to mean BD values between 1.4 and 1.6 g cm^−^³ in sandy soils analyzed through a German mechanical soil data base by Schroeder et al.^35^. In a recent European assessment, bulk density in agricultural soils averaged only 1.26 g cm^− 3,^ with values above 1.5 g cm^−^³ rarely occurring^36^. In the subsoil (D4, D5), discrepancies grew with average values of 1.34 and 1.14 g cm^−^³ at the control site, but 1.66 and 1.68 g cm^−^³ at the Agri-PV site. This increase is most plausibly related to frequent movement of heavy machinery during installation, although contributions from pre-existing subsoil conditions cannot be fully excluded. Ten sampling locations on the Agri-PV transects showed BD values higher than 1.8 g cm^−^³, a level commonly associated with severe mechanical compaction and restricted root growth in coarse-textured soils^37^.

The BD values under the Agri-PV system were considerably above 1.61 g cm^− 3^, a locally established threshold that Frielinghaus et al.^38^ identified as a Level 3 compaction risk in slightly silty sands, linked to yield declines of 10%. Since one third of our sampling points exceeded BD values even above 1.71 g cm^− 3^ across depths, future negative effects on root development and crop performance cannot be excluded^38^. However, the magnitude of yield reduction will depend on crop species, soil texture, climatic conditions, and management. Locally, a study by Hofbauer et al.^39^, in a similar soil type showed that an increased BD of 1.66 g cm^−^³ in 6 cm depth caused by shallow non-inversion tillage restricted nitrogen mineralization and root development, together declining rye yields by 22–43%.

We further assume germination problems and reduced root growth in future crop production under this Agri-PV system, as recently assessed for wheat in a sandy loam at a compaction level of 1.7 g cm^− 3^^40^. This aligns with findings from a global meta-analysis by Zhang et al.^41^, who reported an average bulk density of 1.71 g cm^−^³ and 1.77 g cm^−^³ for compacted coarse-textured soils in 0–30 and 30–40 cm, respectively and found significant yield depressions starting at 1.6 g cm^−^³ for crops like wheat and maize. In loamy sands, bulk densities above ~ 1.6 g cm^− 3^ have been frequently reported to restrict root growth, and values above 1.8 g cm^−3^ may fully inhibit root development (^42^^,43^).

Compaction of this magnitude is particularly concerning in coarse-textured sandy soils, which are highly vulnerable due to their low clay content and weak aggregate stability^44^. In contrast to finer-textured soils, sandy soils lack shrink–swell capacity and exhibit lower biological activity, both of which limit their natural ability to recover from mechanical disturbance^45,46^. These characteristics suggest a comparatively limited natural recovery potential compared with finer-textured soils, meaning that compaction effects may persist for extended periods. The risk was likely increased during Agri-PV installation, as soil moisture levels at that time (Fig. 1) were elevated and reached field capacity in some zones (Fig. 1; Table 1). High soil water content near field capacity is a well-known driver of rapid compaction because water reduces interparticle friction and facilitates particle rearrangement under machinery load^18,47^. While dry soils are less prone to compaction due to increased cohesion, fully saturated soils also become less compactable due to water occupying all pore spaces^48^. Sandy soils and slightly silty sands reach saturation at comparatively low moisture contents of around 30% and field capacity at 20–25 vol.-%^49^ which has been reached in several occasions across depths and transects. This narrow margin between field capacity and saturation makes them especially susceptible to machinery passes during wet periods, particularly under heavy axle loads.

### Penetration resistance confirms mechanical compaction

Penetration resistance (PR) results from the Agri-PV site further support the diagnosis of severe compaction. Mid-depth layers (D3) showed mean PR values of 3.7–4.1 MPa at the P and Ag locations of the Agri-PV site, surpassing the commonly accepted root growth threshold of 2–3 MPa^41^ (Fig. 4). About 70% of crop roots typically occupy the upper 30 cm soil profile^50^, indicating a strong potential for mechanical impedance in those depths with PR above 3 MPa which was the case in many agricultural locations at the Agri-PV site, thus potentially limiting root penetration and restricting root elongation^51^.

This mechanical impedance assumption is consistent with observations from compacted coarse soils globally. In the same meta-analysis by Zhang et al.^41^, PR values under compaction averaged 2.5 MPa, increasing to 5.5 MPa at 30–40 cm in severely compacted sandy sites. In the Agri-PV site, several PR measurements exceeded this value in the intermediate depth. Moderate passes with machinery caused PR to increase by 245% in coarse-textured soils and resulted in an average yield decrease of corn and soybean by 34%, by 16% for barley and by 6% for wheat^52^. However, PR levels were similar in the deeper layers D4 and D5 at the control sites, suggesting comparable levels of soil stress and subsoil compaction. Thus, subsoil compaction cannot exclusively be linked to recent construction activities but may partly reflect pre-existing site conditions, such as pedogenic densification, historical land use, or natural variability in soil composition.

The data also reveals considerable variability of PR in the Ag locations, indicating heterogeneous compaction patterns, likely due to uneven and uncontrolled trafficking during the construction phase and inherent soil properties of texture and moisture. Such heterogeneity in form of patchy compaction zones is typical where traffic concentrates along informal lanes, turning points, or equipment loading areas, rather than being confined to designated tracks^53^. Interpretation of PR should be made with caution, as soil moisture at the time of measurement varied considerably among transects (14–22 vol.-% at 45 cm depth, Fig. 1). Consequently, part of the observed variability in PR likely reflects differences in soil water content in addition to machinery traffic effects.

While PR and BD are often correlated, this relationship is highly dependent on soil moisture and soil type. In our dataset, this relationship was weak and only observed in the topsoil (Fig. 6), likely reflecting spatial heterogeneity and variable water contents at the time of measurements and sampling. While Lardy et al.^54^ found a strong linear relationship under controlled conditions, PR variability increased with lower water content. However, authors collected bulk density data in a range from 1.2 to 1.6 g cm^-3^, leaving little room for interpretation in highly compacted soils. Costantini^55^ cautioned against predicting PR from BD or moisture alone, highlighting the value of measuring both indicators independently but in parallel.

### Avoiding compaction during construction and options for recovery

The severity and spatial extent of compaction observed in this study calls for preventative measures during the Agri-PV construction. In our study, soil compaction occurred around pole bases (due to direct construction impacts), but also across the wider agricultural area between the poles. There was uncontrolled movement of heavy equipment, also under unfavorable weather conditions due to the timing of construction during late autumn and early winter.

To reduce such negative impacts, regulations are being developed. The DIN SPEC 91,434 (2021-05) is the norm for Agri-PV systems and describes the requirements for primary agricultural use in Germany. It ensures that the primary focus of Agri-PV installations remains on agricultural land use. Consequently, soil protection is identified as one of the key principles within this standard, which explicitly outlines measures to prevent soil compaction. These measures include, for example, the use of temporary driving paths and specialized tires, and recommend vehicular traffic to periods under dry conditions (DIN, 2021).

Thus, our study supports the need for more critical pedological construction supervision (PCS). This should involve monitoring of soil protection by qualified personnel throughout planning, execution, and post-construction restoration^56^. Rigorous PCS could provide site-specific recommendations to schedule construction avoiding wet conditions which was not fully possible for the current site due to the very tight project finance- and timeframe imposed by the funding agency. Also, limiting traffic to designated lanes or use of temporary trackways and subsoil protection mats would have been a suitable compaction risk minimization strategy.

Natural processes of freezing/thawing, wetting/drying, and bioactivity can alleviate topsoil compaction^57^. While post-hoc measures for the recovery of compacted soils are available, including mechanical/physical (tillage), biological (earthworms, deep rooting crops) and chemical (lime or manure incorporation) methods^58^, these cannot fully compensate for deep compaction, particularly in sandy soils that lack natural recovery processes^45,46^. Therefore, continuous monitoring of post-construction recovery and future recommendations on subsoiling/deep tillage were put into practice and a combination of mechanical (tillage) and biological (perennial alfalfa cultivation for 2–3 years) was implemented on our study site.

While the risks and mechanisms of soil compaction are well-established in soil science and agronomy, this is not yet the case for companies constructing Agri-PV systems on arable land. When the aim is to increase Agri-PV systems across Europe, greater attention is needed during the planning and implementation phase to avoid soil compaction as much as possible. Policy incentives that emphasize rigid deadlines may inadvertently promote construction activities under sub-optimal conditions, leading to elevated compaction risk. Costs associated with the measures against compaction need to be considered. Immediate post-installation evaluation followed by reassessment three to five years later can provide data on soil structural recovery.

### Study limitations due to experimental design and method selection

Several limitations should be considered when interpreting the observed compaction patterns. First, the inherent spatial heterogeneity of the site, including variations in soil texture, carbon and moisture, may have contributed to part of the variability observed in both bulk density and penetration resistance. However, the consistent increase in bulk density across multiple depths and transects suggests a systematic effect beyond random spatial variability.

Second, the comparability of the control site is limited, as pre-existing soil conditions and management history are not fully known and field edge effects limit its representability. The relatively high subsoil penetration resistance observed at the control site suggests that background soil conditions or prior management may have contributed to baseline compaction levels. This limits the extent to which subsoil differences can be attributed exclusively to construction effects. On the other hand, the small distance between Agri-PV treatment and the control plots ensures most similar soil types for sampling conditions and measured topsoil effects can only be attributed to recent machinery passings which were visually absent from the control site.

Third, sampling accessibility, particularly in compacted subsoil layers, may have constrained measurements. It should be noted that penetration resistance measurements were limited in highly compacted layers due to technical constraints of the penetrologger. As a result, peak compaction levels may be underestimated but were still captured with bulk density assessment.

Finally, while differences between pole and agricultural zones were assessed, the lack of consistent significant differences suggests that traffic intensity and construction processes affected the entire site more uniformly than initially assumed.

## Conclusions

Physical soil properties at the Agri-PV site frequently exceed commonly used compaction thresholds reported for coarse-textured agricultural soils. Bulk density values above 1.67 g cm^-^³ and penetration resistance levels exceeding 3.7 MPa indicate severe mechanical compaction, far beyond levels known to restrict root elongation, reduce crop yields, and impair key soil functions such as infiltration and nutrient cycling. These consequences are well documented in literature and reflect significant risks for agricultural productivity and long-term soil functioning. The weak structural integrity of sandy loams, characterized by low clay content and limited shrink-swell potential, suggests that natural soil recovery processes will be limited at the study site without mechanical intervention.

The inherent heterogeneity of the site limits the extent to which construction-induced effects can be fully separated from background soil variability. However, the magnitude and consistency of observed compaction patterns in both, bulk density and penetration resistance across multiple transects provides independent evidence of construction-related soil compaction. The combined assessment of bulk density and penetration resistance proved valuable, as both indicators complemented each other and helped identify compaction patterns in space and depth despite limitations associated with individual methods.

Our findings strongly justify the implementation of pedological construction supervision in Agri-PV developments, particularly in sandy-soil regions where natural decompaction processes are limited. Proactive soil management strategies should become standard practice, including careful planning of construction timing, load restrictions during wet conditions, and the use of mitigation techniques such as controlled traffic lanes and temporary trackways. Agri-PV projects should incorporate pre-construction soil assessments and post-construction monitoring to better quantify changes attributable to installation and support targeted recovery measures.

To maintain productivity on agricultural land under Agri-PV, controlled traffic farming principles should be prioritized, starting already in the planning phase for construction machinery and to be continued during agricultural management with field machinery. This would help minimize further soil degradation by machinery, ensure long-term land usability, and align Agri-PV development with sustainable land stewardship principles. The usage of light-weight agricultural robotics could enable further soil protection after installation. Further studies across different soil types and Agri-PV configurations are needed to evaluate the transferability of these findings and to improve understanding of soil recovery processes following installation^59^.

## Materials and methods

### Site description

The study site is located on a 0.75 ha field at the research station of the Leibniz Centre for Agricultural Landscape Research (ZALF) in the city of Müncheberg (52.52° latitude, 14.12° longitude, and 63 m above sea level) in Brandenburg, north-east Germany. The long-term (1991–2020) average annual temperature at this site is 9.6 °C, with an average annual precipitation of 552 mm^60^^,61^). The soils are dominated by sand with an average share of 74% and a mean soil organic carbon (SOC) content of 0.95% in the topsoil (4–9 cm, Table 1). The highly heterogeneous soil pattern in this region has been developed from a thin layer of glacial till overlying glaciofluvial sands. According to IUSS (2022), the present soils are mainly classified as Albic, Lamellic Arenosol (Aric). This particular site was historically under fallow from 1989 to 2009, used for poplar clone testing between 2009 and 2018, and under fallow since then. Before the construction started, the field has been mulched, disk-harrowed and ploughed for site and soil homogenization in Spring 2024. Alfalfa (*Medicago sativa*, variety Plato, 12.5 cm row distance and 20 kg/ha seeding density) was planted on April 12th, 2024.

### Agri-PV system

The Agri-PV system was built in autumn 2024 and serves as a pilot project initiated by ZALF for double-purpose crop and energy production in the Brandenburg region. The construction started on October 7th, 2024 and started its operation on December 6th, 2024.

The Agri-PV plant has a total capacity of 583 kWp and a total of 834 bifacial photovoltaic modules, consisting of two different Agri-PV axis tracking systems: a single-axis tracking system with an output of 433 kWp and a dual-axis tracking system with an output of 151 kWp. The single-axis tracking system (2D) consists of a total of four tracking rows with a primary beam height of 4 m and a clearance height of 4.6 m. The spacing between the tracking rows is 10 m. The maximum tilt angles of the tracker are +/- 80°. The single-axis tracking system was installed on an area of 0.6 ha. The dual axis (type altitude–azimuth) tracking system (3D) consists of three solar panel rows with a beam height (primary axis) of 4 m, a clearance height of 4.6 m and a spacing of 16 m. The primary axis is positioned horizontally to the ground, the secondary axis is perpendicular to the primary axis. The system was installed on an area of 0.1 ha. The two tracking systems are separated by an access path of 14 m width, with all tracking rows oriented at approximately 140° (northwest to southeast).

**Fig. 7:**
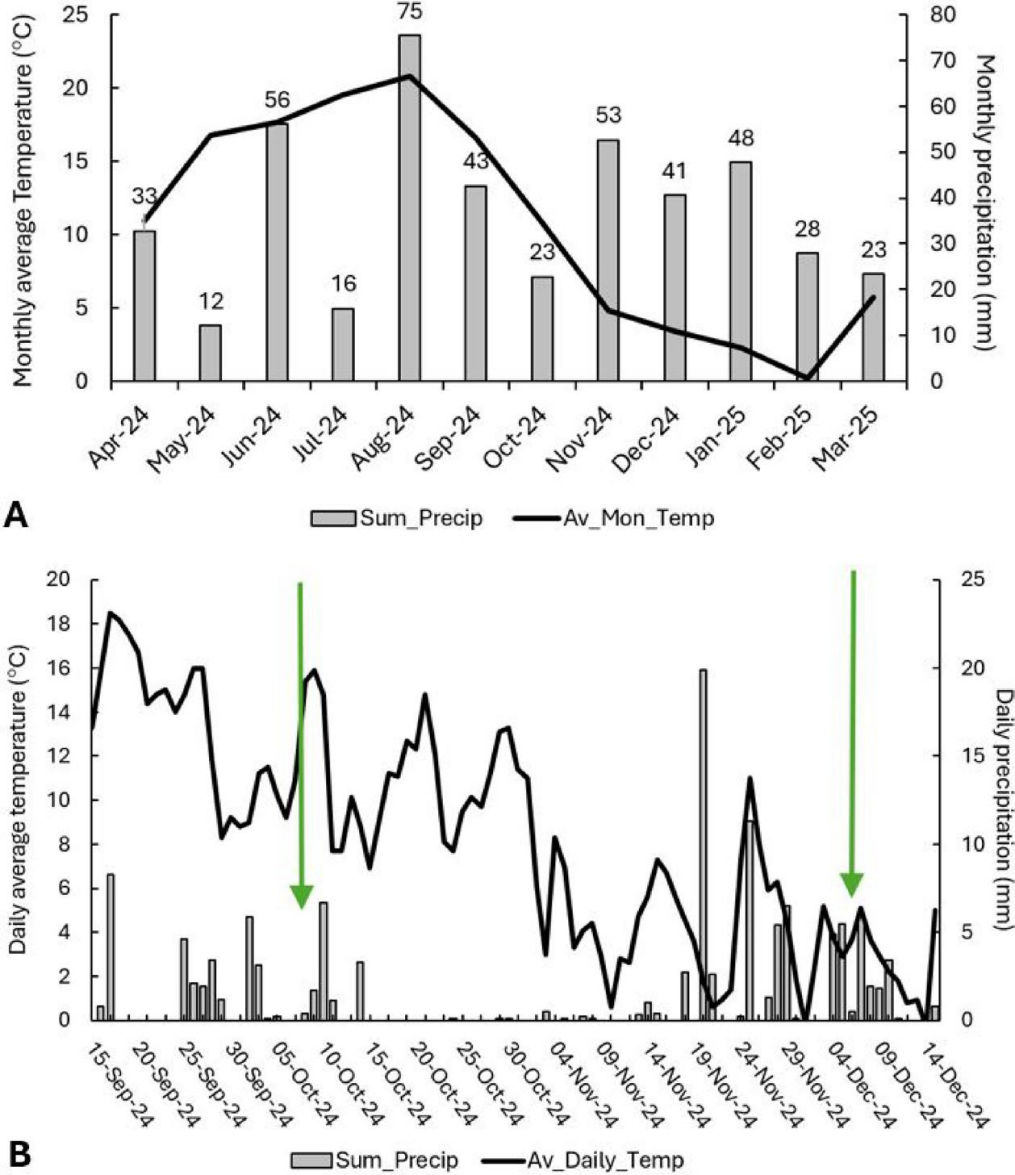
(**A**) Monthly average air temperature and accumulated precipitation since the beginning of the site preparation and (**B**) daily air temperature and precipitation during the Agri-PV system construction period, green arrows indicate the start and end of construction. Data were obtained from a meteorological station 50 m from the study site.

The project faced significant time constraints due to a strict funding deadline, which accelerated the construction process and limited opportunities for soil protection measures. During construction, no specific measures were taken to minimize soil disturbance, as neither traffic restrictions under high soil moisture conditions nor the use of ground protection panels were implied or implemented. The use of ground protection panels was largely avoided due to high rental costs, a one-week delay required for installation and removal, and an increased risk of slipping following rainfall events. The installation took place in fall 2024, which is a period characterized by exceptionally high precipitation levels (Fig. 7). Three weeks before installation started, almost 32 mm of rainfall were recorded. During construction, another 83 mm of rainfall led to challenging conditions. Several heavy-load machines operated on the Agri-PV site, including a scissor lift (4245 kg), front forklift (4220 kg), telescopic forklift Roto (17900 kg), and a crawler excavator Liebherr 924 (23400 kg). Additionally, large semi-trailer trucks for material transport and various smaller chain-driven vehicles such as excavators for digging power cable shafts, machines for anchoring screw foundations, and drill rigs for 3 m boreholes frequently entered the field.

## Experimental design

**Fig. 8:**
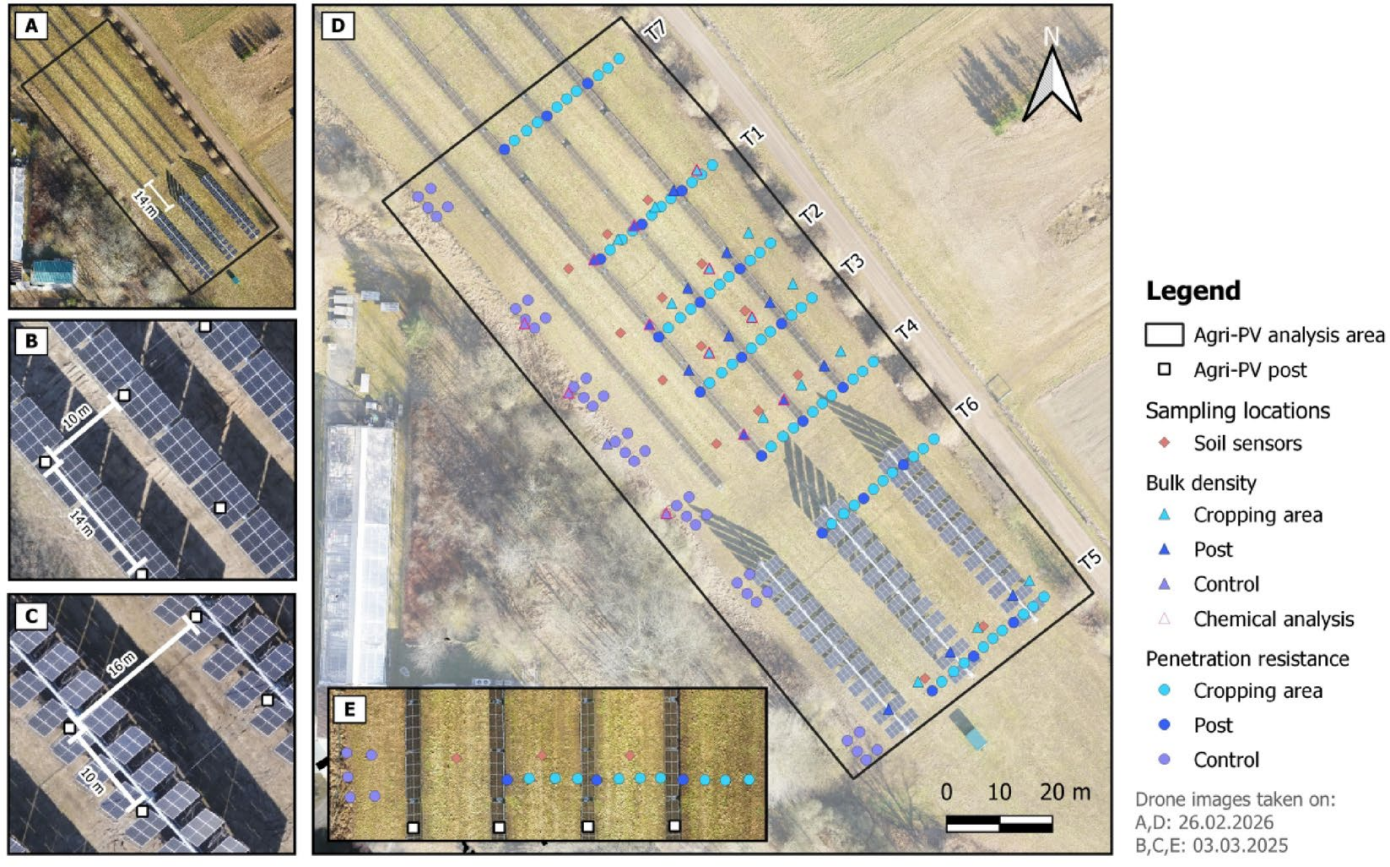
Overview of the experimental site: (**A**) aerial view of the Agri-PV installation, including both the 2D and 3D tracking systems; (**B**) layout and spacing between panel rows and posts in the 2D system; (**C**) layout and spacing between panel rows and posts in the 3D system; (**D**) sampling and transect locations for soil sensors, bulk density, penetration resistance, and chemical soil analyses; and (**E**) example of a transect layout and top view of the reference area (control, Con).

Up to seven measurement transects have been selected perpendicular to the tracking rows to cover the area near the anchored metal posts (P) of three tracking rows and the agricultural area (Ag) in between the panels (Fig. 8). Three points for each location (P, Ag) have been determined along each transect (Fig. 8D&E). Four and five transects (T1-T4, T7), for bulk density and penetration resistance assessment, respectively, were selected in the single-axis 2D system and the distance between the transects was 14 m. Only one and two transects (T5, T6) were established within the 3D system for bulk density and penetration resistance assessment, respectively, because of the small accessible field area and flooded conditions in the trenches during sampling.

Reference areas (Con) served as control for undisturbed soil and were located only few meters outside the main Agi-PV field next to the field edge, still in the agricultural area (Fig. 8E). During sampling, the reference area was a fallow, which is comparable to the original Agri-PV field conditions and previous state before the construction started.

### Data collection and sample analysis

#### Bulk density and soil porosity

Bulk density (BD) was measured using the core method^62^. Soil samples were collected on November 25th, 2024 horizontally via an accessible trench in five transects using a metal core sampler with a known volume (99.57 cm³) at five depths: 4–9 cm (D1), 12–17 cm (D2), 20–25 cm (D3), 45–50 cm (D4) and 70–75 cm (D5). Triplicate samples were collected for each transect near the metal posts (P) and in the agricultural area (Ag). Each sample was carefully extracted to avoid compaction or loss of soil. The samples were then placed in labelled plastic bags and transported to the laboratory. In the laboratory, soil cores were weighed to determine their fresh weight, and then oven-dried at 40 °C for 96 h to obtain the dry weight for gravimetric soil moisture. This procedure has been tested applying the standard bulk density protocol for 10 subsamples dried at 105 °C for 48 h to make sure that constant oven dried mass was achieved, deviations were < 0.4.-vol%. Sample drying at 40 °C was done to reuse samples for further soil chemical analysis at similar depth intervals. From the dried soil samples, visible stones were sorted by hand and weighted for density correction calculations.

The bulk density was calculated using the formula:

1 $$\:BD = \:\frac{{DM}}{V}\:\:$$

where BD = Bulk Density [g/cm³], DM = Mass of oven-dried soil [g] and V = Total volume of the soil sample [cm³].

The corrected bulk density was calculated as follows:

2 $$\:BD_{{corrected}} = \:\frac{{DM - mg}}{{V - \frac{{mg}}{{\rho \:_{s} }}\:}}\:$$

where BD _corrected_ = corrected Bulk Density [g/cm³], mg = Mass of gravel and stones [g] and ρs = assumed density of stones and gravel: 2.65 g/cm^3^ as density of quartz.

Soil porosity was calculated using the following formula:

3 $$\:Porosity\:\left( \% \right) = \:\frac{{100\:(\rho \:{\mathrm{s}} - BD_{{corrected}} )}}{{\rho \:{\mathrm{s}}}}\:\:\:$$

### Penetration resistance

A electronic penetrometer (Penetrologger with GPS, type: 06.15.SA, Royal Eijkelkamp B.V., The Netherlands) was used to conduct measurements in the vertical compaction profile with a depth resolution of 1 cm. Necessary configurations were completed beforehand. A measurement plan was established, involving 119 plots among seven transects: five of them being identical to the transects used for bulk density assessment, and two additional transects have been established for each tracking system to increase the data resolution (Fig. 8D). Each transect covered the sampling locations near the metal posts (P), agricultural area (Ag) between the posts and reference control areas (Con) outside the Agri-PV site, with one penetration conducted per plot. Volumetric soil moisture content was measured simultaneously in the top 5 cm at each plot using an Acclima SDI-12-Sensor Reader-Kit connected to a TDR315L soil moisture sensor. Among the four available cone sizes for the penetrologger, a cone with a cross-sectional area of 1 cm² and an angle of 60° was selected. The penetration speed was set to 2 cm/s, and the penetration depth of the cone was limited to 80 cm, although the maximum depth was not reached in every plot. The device measures soil penetration resistance in MPa by inserting the rod into the soil at 1 cm intervals. Once all measurements have been collected on December 17th, 2024, the penetrologger was connected to a computer, and the data was transferred using Penetro-viewer software (Version 6.08). Subsequently, the data was processed and averaged for five similar depths as bulk density. Negative values were deleted from each measurement and indicated the deepest penetration possible at a certain location. Below that, no further data points have been recorded. They were caused by interrupted measurements due to high soil resistance when no further penetration was possible or due to stones.

### Soil chemical and physical properties

For further site description and determination of small-scale soil heterogeneity, 15 soil samples have been selected from different transects and depths for further texture and chemical analysis (Fig. 8; Table 1). Soil particle size distribution was determined using the PARIO device (Meter Group, USA) in the PARIO Plus measurement mode. The device is placed into a 1 L cylinder with the dispersed soil sample, constantly measuring the suspension pressure at a specific level^63^. In the Plus mode, the measurement is extended by draining and drying an aliquot of the suspension, thereby improving the precision of the measurement^64^. Samples were prepared as follows: Organic material was removed from 20 to 25 g of air-dried soil by adding 50 mL 15-% hydrogen peroxide. For chemical dispersion, 25 mL of tetra sodium pyrophosphate decahydrate (concentration of 44.6 g L^− 1^) was added, and the samples were placed on a shaker for 12 h. Subsequently, the measurement was performed (60 s overhead shaking, draining in Plus mode after 2.5 h). The drained suspension was dried at 105 °C until constant weight to determine dry mass in the drained effluent. The remaining suspension was wet sieved with a sieve stack of 630 μm, 200 μm, and 63 μm to determine the sand content and fractions. Measurement and data evaluation were conducted with the Meter PARIO CONTROL program (software version 1.1.1). For particle density, 2.65 g cm^− 3^ was assumed.

The same subset of soil samples was analyzed for total carbon and nitrogen (CNS928-MLC, Leco Instruments GmbH, according to DIN ISO 13878 and 10694), inorganic and organic carbon content (RC-612, Leco Instruments GmbH, according to DIN ISO 10694), and pH according to DIN ISO 10390 (Table 1).

### Soil moisture

A total of 75 soil sensors (model WET150, Delta-T Devices, United Kingdom) were installed at 25 locations in depths of 45 cm, 75 cm, and 95 cm, distributed along five transects (Fig. 8D). Four of the five transects (T1 to T4) are located in the single-axis tracking system (2D), transect 5 (T5) is located within the dual-axis tracking system (3D). Data presented here only include sensors installed at 45 cm soil depth in the centric agricultural area at positions with undisturbed precipitation exposure between Agri-PV panels. The sensors monitor volumetric soil moisture content, soil temperature, and electrical conductivity in real-time with a 15 min resolution. All sensors started sending data from December 5th, 2024 at 12 am. For data presentation, measured readings were aggregated to an hourly average value.

### Data analysis

Initially, bulk density and soil penetration resistance data were submitted to a quality screening process to identify and remove outliers based on the z-score method (|z| > 3). Then, normality assumptions of the residuals were evaluated by applying the Shapiro-Wilk test. The homogeneity of variances was analyzed by the Levene test. As both assumptions were met, Type II ANOVA was applied, appropriate for unbalanced datasets and different numbers of observations for treatment groups^65^ as the reference area (Con) had a reduced number of observations for both parameters compared to the Agri-PV site. To identify statistically significant effects (*p* < 0.05), Tukey’s HSD (Honestly Significant Difference) post hoc test was performed to identify which groups presented significant differences. Penetration resistance data in the depth profile was expressed as mean ± standard error (SE) and graphically represented in R software (version 4.4.2) with the stat_summary() function of the ggplot2 package^66^. A linear regression model was performed to evaluate the correlation between bulk density and soil penetration resistance. The analyses were performed using R program, version 4.4.2^67^ and Python programming language (Python Software Foundation, 2024), version 3.11.

## Data Availability

The data set "Chemical soil Properties and Soil Compaction measured in 2024 in an Agri-PV System in Brandenburg" is available in the BonaRes repository: 10.4228/zalf-8bd9-7x82.
